# Synergetic effect of a DSP4-induced locus coeruleus lesion and systemic LPS exacerbates substantia nigra dopaminergic neuron loss

**DOI:** 10.1038/s41598-025-33147-8

**Published:** 2025-12-24

**Authors:** Feryal Şimşek, Diana Papová, Emilia Henriksson, Sonia Olmedo-Díaz, Marcus Blyberg, Roine El-Habta, Ana Virel, Sara af Bjerkén

**Affiliations:** 1 Department of Medical and Translational Biology, Umeå University, Linnéus väg 9, Umeå, 90736 Sweden; 2 Department of Clinical Sciences, Neurosciences, Umeå University, Daniel Naezéns väg, Umeå, 90737 Sweden

**Keywords:** Parkinson’s disease, Locus coeruleus, Neuroinflammation, Noradrenaline, Neurology, Neuroscience

## Abstract

Parkinson’s disease (PD) involves progressive degeneration of dopaminergic neurons in the substantia nigra (SN) and noradrenergic neurons in the locus coeruleus (LC). Noradrenaline (NA), produced by LC neurons, has anti-inflammatory properties and seems to support SN dopaminergic neuron survival. This study used a dual-hit approach to examine how noradrenergic loss affects SN vulnerability to induced inflammation in Sprague-Dawley rats. LC denervation was produced with [N-(2-chloroethyl)-N-ethyl-2-bromobenzylamine] (DSP4), a selective LC noradrenergic neurotoxin, and a systemic inflammatory challenge was induced with a subthreshold dose of lipopolysaccharide (LPS). Behavioral assessments were performed, and neuronal populations were quantified by unbiased stereology. DSP4 or LPS alone reduced tyrosine hydroxylase-positive neurons in both LC and SN, with the DSP4-induced SN loss linked to NA depletion. DSP4 and LPS co-treatment produced greater dopaminergic degeneration in the substantia nigra than either treatment alone, suggesting an additive effect. Furthermore, immunoanalytical analysis using ELISA demonstrated altered cytokine levels in both SN and LC. Taken together, the results support a dual-hit theory, in which additive insults act to push the system toward a tipping point, beyond which SN dopaminergic cell loss becomes extensive.

**Supplementary Information:**

The online version contains supplementary material available at 10.1038/s41598-025-33147-8.

## Introduction

The etiology of Parkinson’s disease is considered multifactorial, with dopaminergic cell loss in the midbrain substantia nigra (SN) likely arising from the convergence of several distinct insults. Beyond this hallmark pathology, there is also well-documented evidence of noradrenergic neuronal loss in the locus coeruleus (LC) of the brainstem^1^. The temporal relationship between dopaminergic and noradrenergic degeneration remains unclear; however, the Braak theory provides a framework for understanding the progressive nature of Parkinson’s disease. It suggests that the disease follows a sequential pattern, with alpha-synuclein aggregation beginning in the olfactory bulb and lower brainstem before spreading to the midbrain and eventually the cortical regions^1^. It emphasizes the heterogeneity of clinical Parkinson’s disease presentations, from early-stage symptoms, such as anosmia and gastrointestinal dysfunction, to late-stage manifestations, including cognitive impairment and hallucinations. The hypothesis implies that degeneration of the noradrenergic LC in the brainstem precedes damage to the dopaminergic neurons in the SN. This idea is supported by research emphasizing the crucial role of an intact noradrenergic system in sustaining the survival of nigral dopaminergic neurons^2^. Interestingly, LC noradrenergic degeneration is documented in both Alzheimer’s and Parkinson’s disease^3^, and the extent of neuronal loss in the LC correlates positively with amyloid plaque and tangle burden in Alzheimer’s disease^4^. In transgenic mouse models of Alzheimer’s disease, LC lesions exacerbate amyloid accumulation^5^. Moreover, oxidized noradrenaline (NA) has been found to disaggregate alpha-synuclein fibrils into oligomers^6^, suggesting a role for NA in amyloid formation in neurodegenerative diseases.

NA intricately modulates a myriad of physiological processes spanning arousal, attention, mood regulation, and stress response. However, emerging evidence implicates dysregulation of NA signaling in the pathogenesis of Parkinson’s disease, with alterations in NA levels observed in both preclinical models and clinical cohorts^3,7,8^. This dysregulation contributes not only to motor dysfunction but also to the exacerbation of non-motor symptoms^9^.

Neuroinflammation is recognized as a central feature of Parkinson’s disease pathology^10^. Beyond its classical role as a neurotransmitter, NA also exerts significant anti-inflammatory and neuroprotective effects. It mitigates oxidative stress-induced neuronal damage and influences neurotrophic factor signaling, thereby supporting neuronal survival^11^. Notably, elevated levels of pro-inflammatory cytokines such as TNF-α and IL-1β have been detected in the brains of individuals with Parkinson’s disease, reflecting a heightened inflammatory state^12,13^. These findings underscore the multifaceted role of NA in modulating the inflammatory environment associated with neurodegeneration in Parkinson’s disease.

Microglia, as resident immune cells of the central nervous system, play a pivotal role in surveilling the brain microenvironment, maintaining homeostasis, and triggering inflammatory responses in the case of injury or insult. However, aberrant microglial activation and dysregulated neuroinflammation emerge as hallmarks of Parkinson’s disease pathology, perpetuating neuronal demise and exacerbating disease progression^14^. In this context, the modulation of microglial activation by NA signaling represents a promising avenue for therapeutic intervention, with implications for mitigating neuroinflammatory cascades and preserving neuronal integrity in Parkinson’s disease^15^.

Despite substantial research on Parkinson’s disease, critical gaps remain in our understanding of its etiopathogenesis and progression, including the contribution of LC noradrenergic system degeneration. This underexplored aspect of Parkinson’s disease pathology warrants further investigation, as emerging evidence suggests that LC-NA dysfunction may play a pivotal role in both motor and non-motor symptomatology. Addressing this knowledge gap is essential to advance our mechanistic understanding of Parkinson’s disease.

Advancing age is the most significant risk factor for Parkinson’s disease. A gradual loss of nigral dopaminergic neurons occurs as part of normal aging; however, when this process becomes accelerated or pathologically altered, the likelihood of developing Parkinson’s disease increases. The dual-hit hypothesis extends this concept by proposing that an initial insult, including early involvement of peripheral or brainstem structures and loss of noradrenergic neurons, creates a compromised system. When a subsequent, secondary insult occurs, the combined burden may exceed a critical threshold, triggering the clinical manifestation of Parkinson’s disease.

The toxin-based [N-(2-chloroethyl)-N-ethyl-2-bromobenzylamine] (DSP4)-rat model can be used to model and study noradrenergic denervation in vivo. DSP4 is a neurotoxin specific for the LC noradrenergic system^16^. Using this model, a direct link between noradrenergic denervation and long-term dopaminergic cell loss in the SN, observed 6-months post noradrenergic lesion, has been established. Additionally, increased nigral neuroinflammation, marked by microglial activation, has been seen following noradrenergic denervation^2^, further highlighting the role of noradrenergic signaling in neuroinflammatory responses. Noradrenergic depletion has been implicated in increased susceptibility of nigral dopaminergic system to inflammatory insults^17^, but its long-term consequences remain poorly understood. Short-term lesion studies have reported minimal DSP4 effects on SN neurons^18^, leaving open the question of whether chronic disruption of noradrenergic signaling is what influences their vulnerability. Sex differences in neuroinflammatory responses may further shape this relationship. In male rodents, LC degeneration combined with immune challenges such as LPS may provoke near-maximal inflammatory responses, potentially producing a ceiling effect that obscures modulatory influences^19,20^. In contrast, females often display more moderate or attenuated neuroimmune activation following lipopolysaccharide (LPS) induced inflammation^21–23^, offering a wider dynamic range in which to detect subtle effects of noradrenergic loss on dopaminergic vulnerability after LPS induced inflammation.

In this project, we utilize the DSP4-rat model of noradrenergic denervation in female Sprague-Dawley to further investigate the role of NA on the survival of SN dopaminergic neurons, and to elucidate how the NA system influence neuroinflammation. To this end, we employ a dual-hit paradigm, which posits that neurodegeneration arises when sequential insults overwhelm the brain’s compensatory capacity. In our model, the first insult is the loss of NA innervation to the SN due to LC degeneration, thereby removing a critical neuroprotective influence. The second insult is systemic administration of lipopolysaccharide (LPS), which induces a robust inflammatory response^24,25^. We hypothesize that this combination accelerates dopaminergic neurodegeneration in the SN.

## Methods

### Animals

Female Sprague-Dawley rats (*N* = 93) 6–8 weeks old, weighing 150–180 g at the time of purchase (Taconic, Ry, Denmark), were utilized in this study. The animal care and experimental procedures were carried out in accordance with the Directive 2010/63/EU of the European Parliament and of the Council on the protection of animals used for scientific purposes and was also approved by the Northern Swedish Committee for Ethics in Animal Experiments. The study is reported in accordance with the ARRIVE guidelines. All efforts were made to minimize animal suffering, and animals were housed under a 12:12 h light-dark cycle with access to food pellets and water *ad libitum*.

### Study design

#### Effects of a dual-hit approach on long-term nigral dopaminergic cell survival

**Fig. 1:**
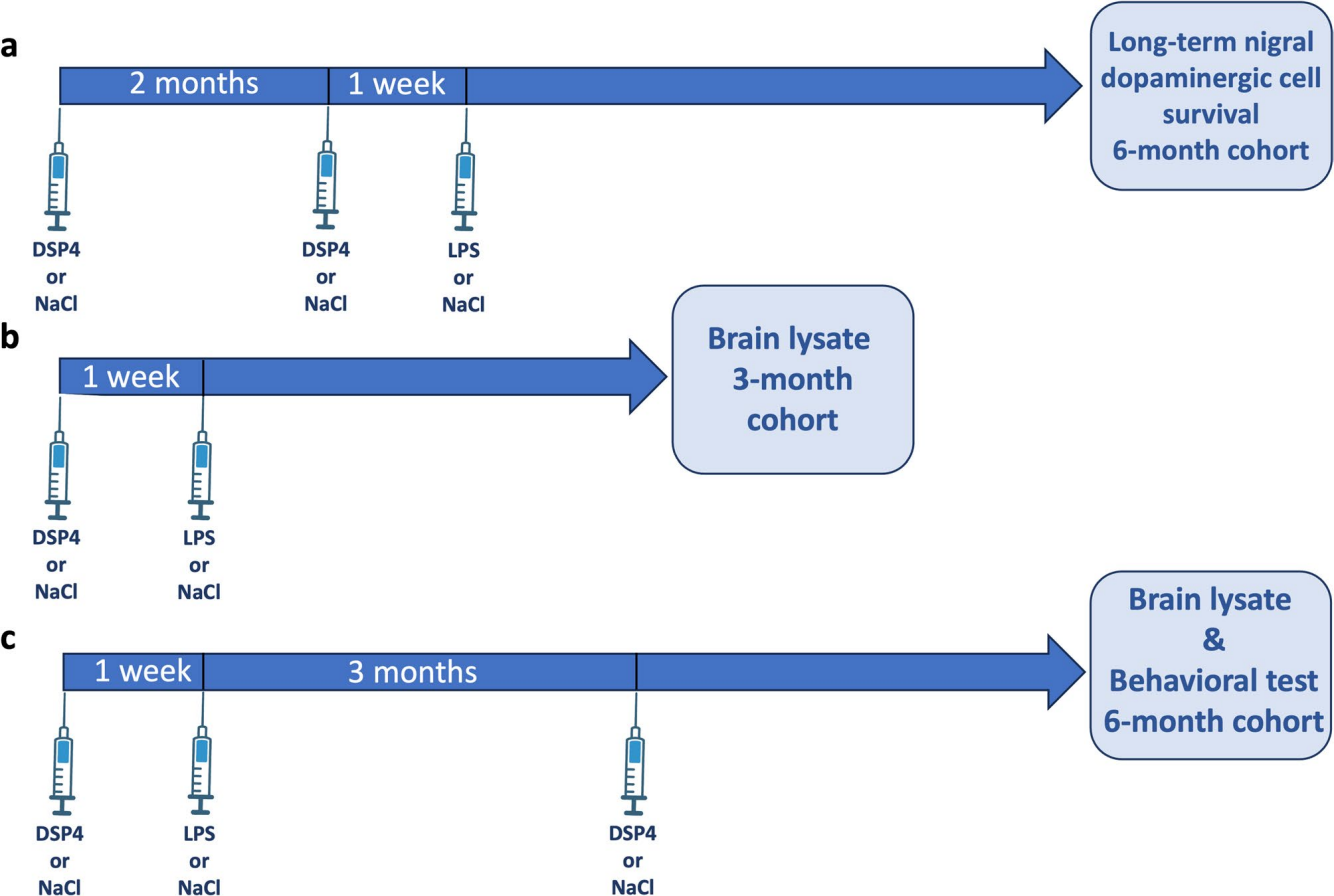
Illustration of the injection protocols and study design. Dual-hit stereology cohort. (**a**). Subjects (*n* = 29) received two consecutive intraperitoneal injections of DSP4 (50 mg/kg; administered 2 months apart) to induce a stable noradrenergic lesion (as described in af Bjerkén et al.^2^), followed by a single dose of LPS (2.5 mg/kg body weight) one week after the final DSP4 injection. At 6-months, the animals were euthanized, brains dissected, and the tissue processed for immunohistochemistry and cell quantification using unbiased stereology. Neuroinflammatory response cohort. 3-months group (**b**), 6-months group, (**c**). Subjects (*n* = 64) were divided into two groups and administered intraperitoneal injections of either N-ethyl-2-bromobenzylamine (DSP4; 50 mg/kg) or saline. One week later, to induce a neuroinflammatory response, half of the subjects received intraperitoneal injections of LPS (2.5 mg/kg body weight), while the remaining half were given saline. Three months later, a subset of the rats (*n* = 32, designated as 6-months point subjects, (**c**) received booster DSP4 injections. At the conclusion of each experimental period, the animals were euthanized, and their brains were rapidly dissected on ice for subsequent biochemical analyses, including cytokine array and ELISA. Behavioral testing was performed on a sub-set of animals at 3 and 6 months (**c**). (Adapted and retrieved from https://app.biorender.com/biorender-templates.).

To test the proposed dual-hit hypothesis – specifically, whether nigral dopaminergic neurons are more vulnerable to inflammatory insults in the absence of noradrenergic input – a cohort of animals were administered DSP4 (50 mg/kg); two injections with a two-month interval between doses to induce a stable noradrenergic lesion^2^. DSP4 injections were followed by a single dose of LPS. The timing was selected to ensure that the inflammatory challenge occurred when the brains were already NA-compromised. Control animals were injected with saline (0,9% NaCl) instead of DSP4 and/or LPS. At 6-months, the animals were euthanized, brains dissected, and the tissue processed for immunohistochemistry and cell quantification using unbiased stereology. Groups were randomized as follows: DSP4/NaCl (onward “DSP4”), *n* = 7; NaCl/NaCl (onward “NaCl”), *n* = 5; DSP4/LPS (onward “DSP4-LPS”), *n* = 11; NaCl/LPS (onward “LPS”), *n* = 6. The study design is illustrated in Fig. 1a.

#### Neuroinflammatory response

In addition to the animals used to test the dual-hit hypothesis, a separate cohort was designated to investigate the progression of neuroinflammatory processes over time, specifically at 3- and 6-months. To this end, we adopted an alternative injection schedule in which neuroinflammation was induced by administering LPS one week after the initial DSP4 injection in both the 3-month and 6-month groups. This approach allowed us to assess the neuroinflammatory response over time, including in the short-term (3-month) group, within the dual-hit paradigm. For the 6-month group, to keep a stable NA denervation, a booster dose of DSP4 (50 mg/kg) was administered three months after the initial injections. Control animals received saline injections instead of DSP4 and/or LPS. At the end of the respective experimental periods, the animals were euthanized, and their brains dissected on ice for subsequent biochemical analyses using a cytokine array and ELISA. Behavioral tests were conducted 3 and 6 months before euthanasia, following prior training of the animals. Animals were randomized as follows: 3-months: DSP4/NaCl (onward “DSP4”), *n* = 8; NaCl/NaCl (onward “NaCl”), *n* = 8; DSP4/LPS (onward “DSP4-LPS”), *n* = 8; NaCl/LPS (onward “LPS”), *n* = 8; 6-months: DSP4/NaCl (onward “DSP4”), *n* = 8; NaCl/NaCl (onward “NaCl”), *n* = 8; DSP4/LPS (onward “DSP4-LPS”), *n* = 8; NaCl/LPS (onward “LPS”), *n* = 8. The study design is illustrated in Fig. 1b-c.

### Noradrenergic denervation

Selective denervation of the LC noradrenergic nerve terminals was performed via systemic [N-(2-chloroethyl)-N-ethyl-2-bromobenzylamine] (DSP4, 50 mg/kg; Sigma-Aldrich/Merck, Darmstadt, Germany) injections^2,16,26^. The toxin was dissolved in 0,9% NaCl and thereafter immediately injected intraperitoneally. To sustain a stable noradrenergic denervation throughout the experiment, animals were given an additional booster dose of DSP4 (50 mg/kg).

### Lipopolysaccharide-induced neuroinflammation

Neuroinflammation was triggered by a single dose of lipopolysaccharide (LPS; E.coli O111:B4, Calbiochem/Merck, Darmstadt, Germany; 2.5 mg/kg body weight), systemically injected intraperitoneally.

### Tissue processing for immunohistochemistry

Animals were euthanized at 6 months using pentobarbital, and transcardial perfusion was performed with calcium-free Tyrode’s solution followed by 4% paraformaldehyde in 0.1 M phosphate buffer. The brains were dissected, post-fixed in 4% paraformaldehyde for 4 h, and subsequently rinsed in a solution of 10% sucrose and 0.01% NaN₃ in 0.1 M phosphate buffer. For sectioning, brain tissue was frozen using CO₂, and 40 μm sections were prepared using a cryostat and mounted onto chrome-alum gelatin-coated glass slides. Sections were incubated for 48 h at 4 °C with primary antibodies against tyrosine hydroxylase (TH; rabbit anti-TH, 1:300; Millipore, Temecula, CA, USA or mouse anti-TH, 1:1500; Immunostar, Hudson, WI, USA). After primary incubation, sections were incubated with secondary antibodies for 4 h at room temperature. These included Alexa Fluor^®^-conjugated antibodies: A594 goat anti-mouse (1:500; Molecular Probes, Leiden, The Netherlands) and A488 goat anti-rabbit (1:500; Molecular Probes, Leiden, The Netherlands). All antibody dilutions were prepared in 1% Triton X-100. Prior to secondary antibody incubation, sections were blocked with 5% goat serum in phosphate-buffered saline (PBS) for 15 min at room temperature. Between incubation steps, sections were rinsed three times in PBS.

### Unbiased stereological cell counting using the optical fractionator

Quantification was carried out on 40 μm serial sections encompassing the LC (1:5) and SN (1:8) using the optical fractionator method^27,28^. A fluorescence microscope (Olympus BX61, Olympus) equipped with a motorized x-y-z stage and Stereo Investigator software (MBF Bioscience, Williston, VT, USA) was used for the analysis. The regions of interest were delineated at low magnification using a 10x objective lens. For SN, cell counting was confined to TH-immunoreactive (-ir) neurons located in the pars compacta and pars lateralis. The dimensions of the counting frame were set at 100.5 μm (width, x), 75.67 μm (height, y), and 20 μm (depth, z), with a sampling grid size of 180 × 180 μm. For LC, the counting area targeted noradrenergic neurons in the A6 region. The counting frame measured 61.55 μm (x), 46.34 μm (y), and 20 μm (z), with a sampling grid of 90 × 90 μm. Using a systematic random sampling approach, cells were counted at high magnification with a 60x objective lens and a numerical aperture of 1.4. All quantifications were performed blinded. As the starting section from the caudal end varied between brains, a random systematic sampling strategy was maintained throughout.

### Behavioral testing

#### Motor function

To evaluate motor coordination in the animals, an accelerating rotarod apparatus (Med Associates Inc., USA) was utilized. The setup featured a rotating rod that increased speed from 4 to 40 revolutions per minute (rpm) over a 5-minute period. Each trial commenced once the animal was placed on the rod and ended either when the rat fell or after a maximum duration of 300 s. Three successive trials were conducted, each separated by a 15-minute rest period. The average time spent on the rotarod was then calculated. Prior to testing, the rats were trained on three separate occasions.

#### Anxiety-like behaviors

Anxiety-like behavior were assessed using the elevated zero maze and marble-burying test^29^. The elevated zero maze, a modification of the elevated plus maze model, consists of an elevated annular platform with two enclosed and two open quadrants, allowing uninterrupted exploration. Supposably, the subjects with anxiety-like behaviors prefer to stay at the enclosed quadrants rather than the open quadrants. Subjects were recorded for 10 min. The first 4 min were used to explore the surroundings, and the time spent in the quadrants during the last 6 min was measured. The marble-burying test was conducted by placing the animals in a standard cage with 8 cm of wood chip bedding and 10 evenly spaced marbles for a duration of 10 min. When anxious, animals tend to bury more marbles. This is thought to reflect hypervigilance or an attempt to “neutralize” a perceived threat or discomfort. The number of buried marbles was counted, with a marble considered buried if at least two-thirds of it was covered by bedding. Marbles that were reburied were also included in the count.

### Brain cell-lysate preparation

At the experimental endpoints (3- and 6-months), animals were euthanized, and their brains were freshly collected. The regions of interest, including the LC, SN, and striatum, were dissected on ice for post-mortem immunoanalysis. The tissues were preserved at −80 °C, and cell lysates were prepared prior to the analysis. Tissue samples were homogenized in RIPA buffer (Thermo Fisher Scientific) supplemented with protease inhibitors (Sigma-Aldrich). After 30 min on ice, the homogenate was centrifuged at 13,400 x *g* for 15 min at 4 °C to remove cellular debris. Total protein was quantified using Bradford Protein Assay Kit (Thermo Fisher Scientific) according to the manufacturer’s protocol. The protein concentrations of each sample were used to standardize the following ELISA results.

### Cytokine array

Pooled lysates from the LC, SN, and striatum of 3-month subjects were analyzed using the Proteome Profiler Rat XL Cytokine Array (R&D Systems) to assess a broad range of cytokines, including microglial activity markers: IL-1β, Interleukin-4 (IL-4) and TNF-α (data not shown).

### Enzyme-linked immunosorbent assay

Based on the cytokine array results and a review of cytokine markers associated with various microglial activation states^30^, IL-1β, TNF-α, and IL-4 were selected for further analysis. The concentrations of the proinflammatory cytokines IL-1β and TNF-α, as well as the anti-inflammatory cytokine IL-4, were quantified using ELISA kits specific for rat TNF-α, IL-1β and IL-4 (Thermo Fisher Scientific).

### Data analysis and statistics

The authors were blinded to the experimental protocol while performing the experiments and the statistical calculations. The statistical analyses and graph generation were performed in GraphPad Prism (version 7.05, GraphPad software, LCC). An a priori power analysis was conducted using an online sample size calculator (ClinCalc.com) to determine the required sample size. The analysis was based on a medium effect size (Cohen’s *d* = 0.50), an alpha level of 0.05, and a desired power of 0.80. The expected effect size was derived from findings in previous studies. Data were first tested for normality using the Shapiro–Wilk test. For data that met the assumptions of normality and homogeneity of variance, two-way ANOVA was performed, followed by post hoc analysis using Tukey’s multiple comparisons test. For non-normally distributed data, the Kruskal–Wallis test followed by Dunn’s multiple comparisons test was used. Results from parametric tests are presented as mean ± standard deviation (SD), while results from non-parametric tests are reported as median and interquartile range (IQR). A *p*-value < 0.05 was considered statistically significant.

## Results

### DSP4 and LPS-induced loss of noradrenergic neurons in the locus coeruleus

**Fig. 2:**
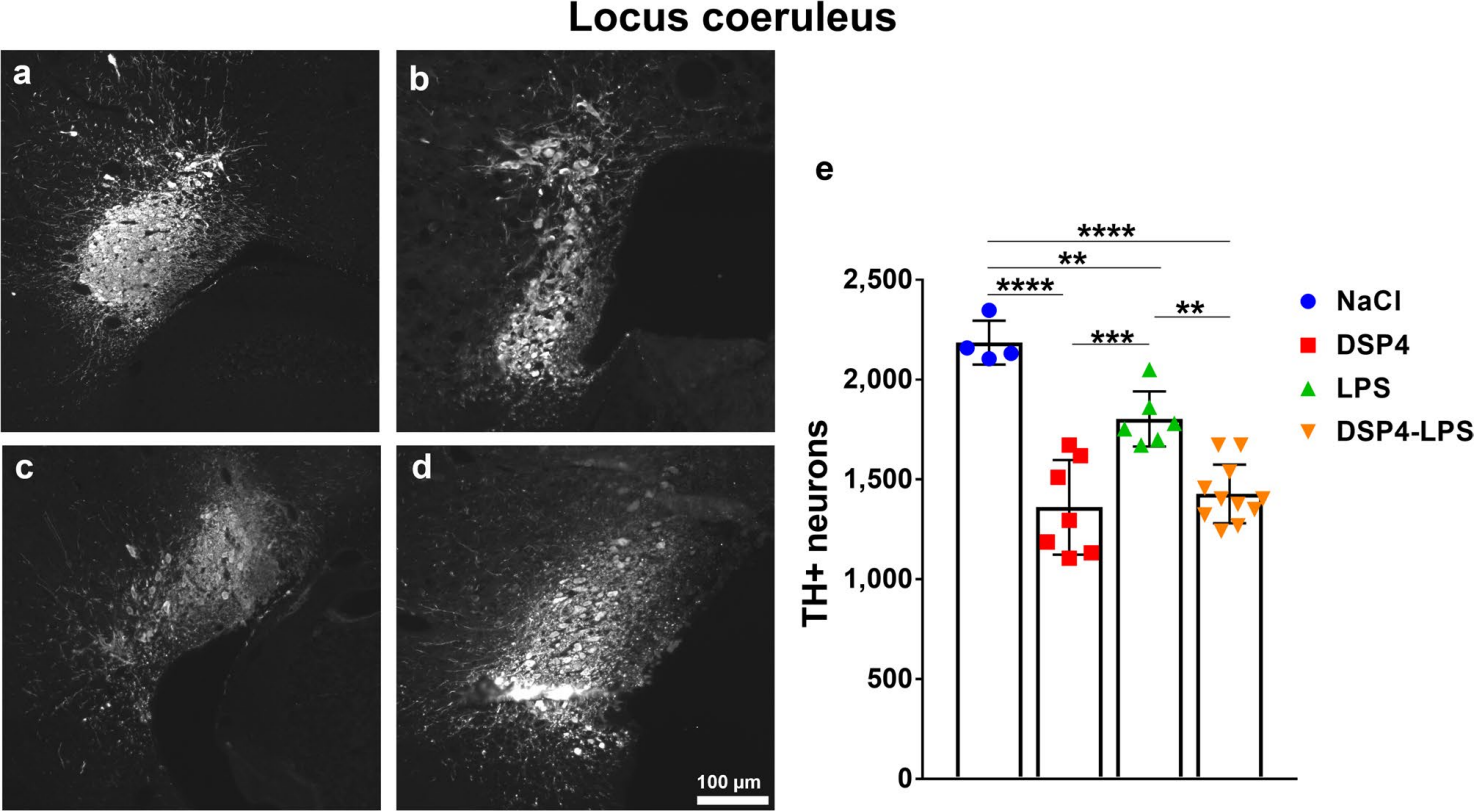
Noradrenergic neuron loss in the locus coeruleus. Photomicrographs of TH-immunoreactive (TH-ir) neurons in the locus coeruleus from animals treated with NaCl (**a**), LPS (**b**), DSP4 (**c**), and DSP4-LPS (**d**), 6 months after the first injection (scale bar = 100 μm). Stereological quantification of TH-ir neurons using the optical fractionator method demonstrated significant reductions in TH-ir neuron numbers in DSP4, LPS, and DSP4-LPS animals compared to NaCl controls. Additionally, DSP4 produced a greater reduction in neuronal numbers compared to an LPS insult, with no statistical significance between DSP4 and DSP4-LPS (**e**). NaCl (*n* = 4), DSP4 (*n* = 7), LPS (*n* = 6), DSP4-LPS (*n* = 11). Bars represent mean ± SD. **p < 0.01, ***p < 0.001, ****p < 0.0001.

The effects of DSP4-induced noradrenergic denervation and LPS-induced inflammation on tyrosine hydroxylase-immunoreactive (TH-ir) neurons were evaluated in the LC (Fig. 2a-d), using unbiased stereological quantification. Two-way ANOVA revealed significant main effects of both DSP4 treatment (*F*₁,₂₄ = 77.76, *p* < 0.001) and LPS administration (*F*₁,₂₄ = 5.36, *p* = 0.029), each associated with reduced TH-ir cell counts (Fig. 2e). However, a significant interaction between DSP4 and LPS (*F*₁,₂₄ = 10.88, *p* = 0.003) complicates the interpretation of the main effects, suggesting that one treatment influences the effect of the other. To clarify this, Tukey’s post hoc analysis was performed, revealing significant reductions in TH-ir neurons in all groups compared to NaCl controls (DSP4, *p* < 0.0001; LPS, *p* = 0.0091; DSP4-LPS, *p* < 0.0001). The combination of DSP4-LPS showed greater loss than LPS only (*p* = 0.0011), while no difference was observed between DSP4-LPS and DSP4 only (*p* = 0.8444). DSP4 had fewer neurons than LPS (*p* = 0.0005), suggesting DSP4 to have a dominant effect. In all, the results suggest that DSP4 and LPS influence the LC separately, with no synergistic impact.

### Reduced nigral dopaminergic neurons following a dual-hit

**Fig. 3:**
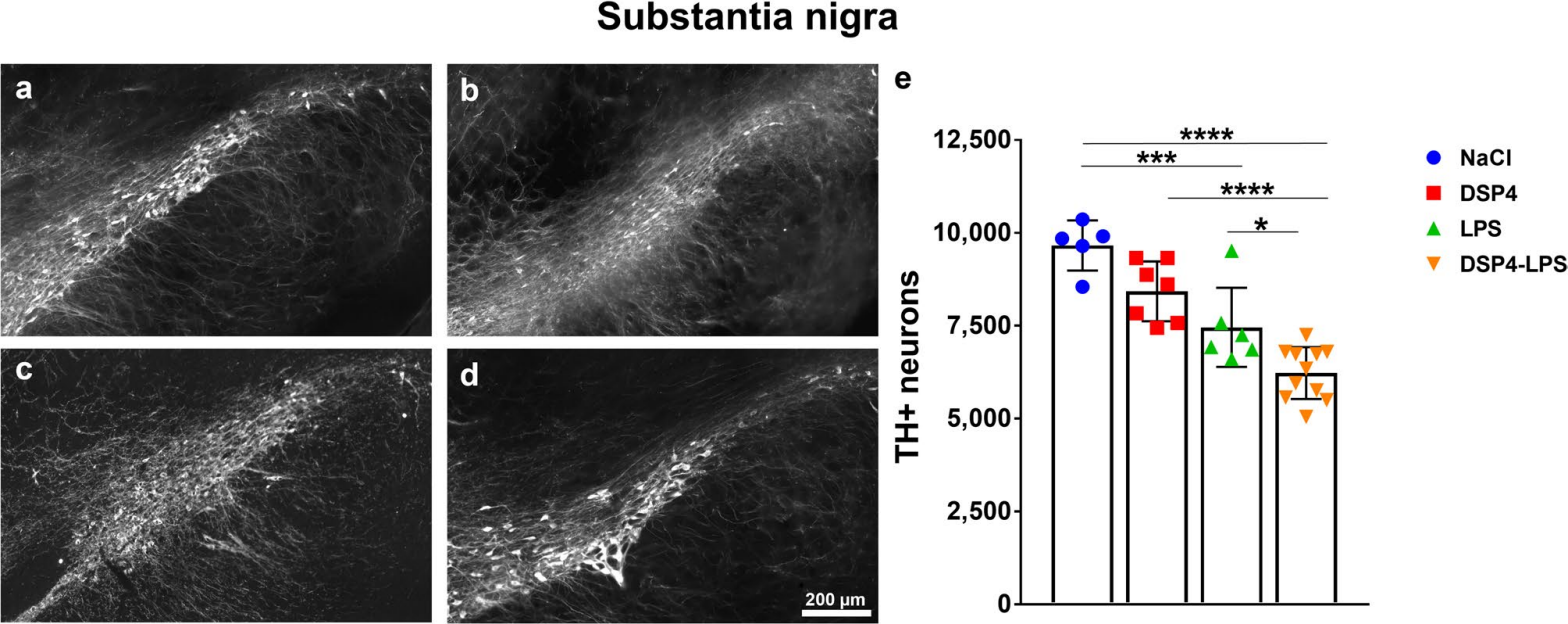
Dopaminergic neuron loss in the substantia nigra. Photomicrographs of TH-ir neurons in the substantia nigra from animals treated with NaCl (**a**), LPS (**b**), DSP4 (**c**), and DSP4-LPS (**d**), 6 months after the first injection (scale bar = 200 μm). Stereological quantification revealed that both LPS and DSP4-LPS groups had significantly fewer TH-ir neurons compared to NaCl controls. Furthermore, neuronal loss was significantly greater in the DSP4-LPS group than in animals treated with either DSP4 or LPS alone (**e**). NaCl (*n* = 5), DSP4 (*n* = 7), LPS (*n* = 6), DSP4-LPS (*n* = 11). Bars represent mean ± SD. *p < 0.05, ***p < 0.001, ****p < 0.0001.

In the SN (Fig. 3a-d), two-way ANOVA revealed significant main effects of both DSP4-induced noradrenergic denervation (*F*₁,₂_5_ = 15.56, *p* = 0.0006) and LPS administration (*F*₁,₂_5_ = 49.72, *p* < 0.0001), (Fig. 3e). No significant interaction between DSP4 and LPS was found (*F*₁,₂_5_ = 0.00006, *p* = 0.9938), indicating additive rather than synergistic effects. Post hoc Tukey’s comparisons showed a significant reduction in TH-ir neurons in the LPS group (*p* = 0.0007) and a trend towards reduction in the DSP4 group (*p* = 0.0667) relative to NaCl controls. Furthermore, the DSP4-LPS group showed a significantly greater loss of TH-ir neurons compared to the DSP4 group (*p* < 0.0001) as well as significant reduction compared to the LPS-only group (*p* = 0.0285). In all, although the LPS-insult itself significantly reduced the neurons in the SN, the results indicate substantial nigral dopaminergic cell loss following a dual-hit.

### Noradrenergic lesioning exacerbates anxiety-like behavior over time

Anxiety-like behavior was assessed using the elevated zero-maze and marble burying tests.

**Fig. 4:**
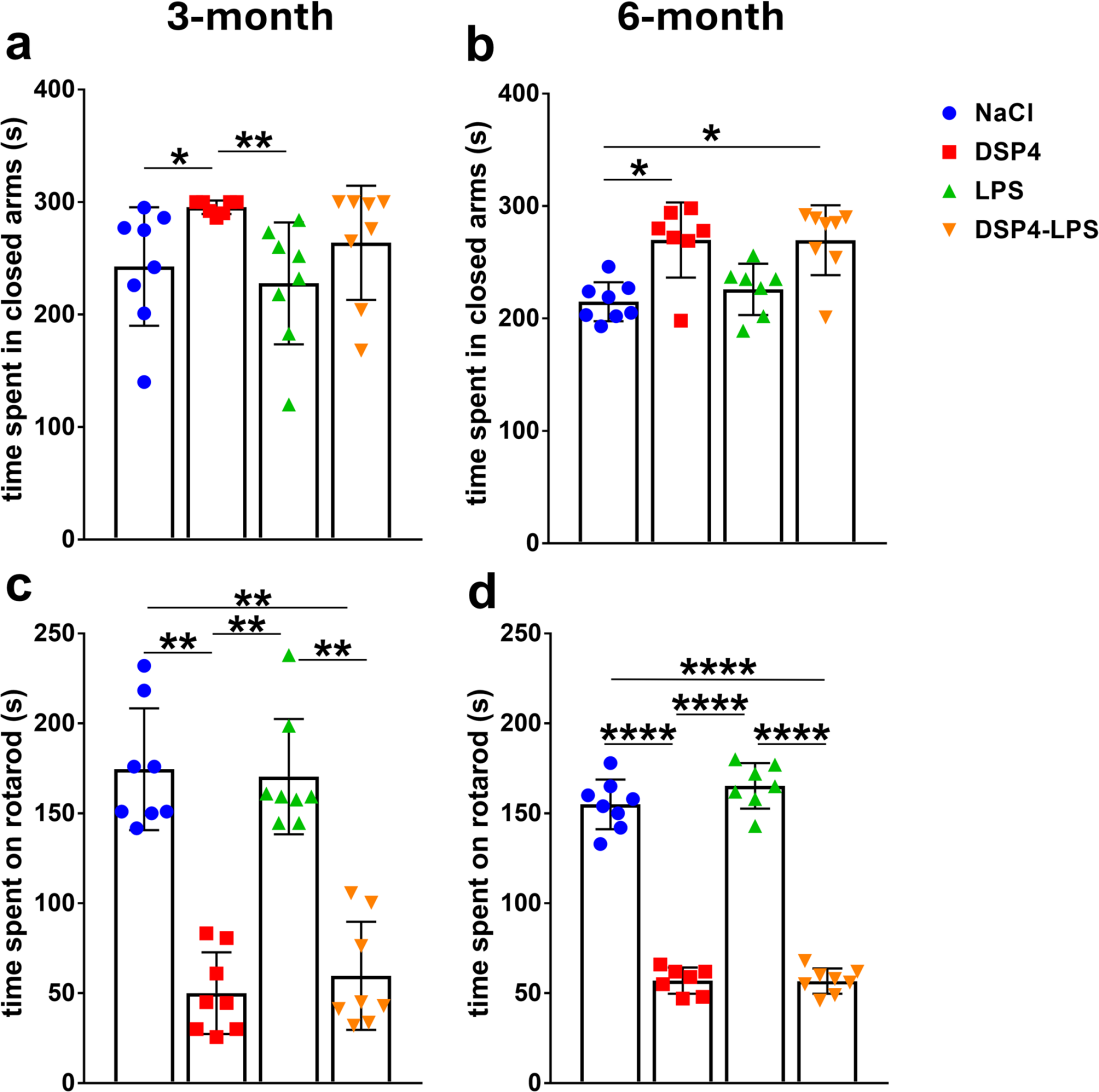
Behavioral assessments. Elevated zero-maze. At 3 months, group differences were observed, with reduced open-arm time/more time in closed-arm in DSP4-treated animals compared to both NaCl and LPS groups (**a**). At 6 months, DSP4 and DSP4-LPS groups spent less time in the open arms/more time in closed arm compared to NaCl controls (**b**). Motor performance. Rotarod test revealed a significant main effect of DSP4 treatment at both 3 (**c**) and 6 months (**d**), indicating persistent motor impairment. NaCl (*n* = 8), LPS (*n* = 8), DSP4 (*n* = 8), DSP4-LPS (*n* = 8). Bars represent mean ± SD. *p < 0.05, **p < 0.01, ****p < 0.0001.

In the zero-maze test at 3 months, Kruskal–Wallis revealed a significant group effect (Kruskal–Wallis *H*_3_ = 12.7; 4 groups, *n* = 31, *p* = 0.0053). Dunn’s multiple comparisons test showed that the DSP4 group spent significantly more time in the closed arm compared with both the NaCl group (*p* = 0.0455) and the LPS group (*p* = 0.0058). Other comparisons were not statistically significant (*p* > 0.05) (Fig. 4a). At 6 months, differences between the groups remained significant (Kruskal–Wallis *H*_3_ = 12.89; 4 groups, *n* = 30; *p* = 0.0049). Dunn’s test indicated that both DSP4 and DSP4 + LPS groups spent significantly more time in the closed arms compared to NaCl (*p* = 0.0359 and *p* = 0.0247, respectively). No other group differences were statistically significant (*p* > 0.05) (Fig. 4b).

Repetitive digging behavior was evaluated at 3 and 6 months using the marble burying test. At 3 months, the Kruskal–Wallis test showed a significant effect of treatment (Kruskal–Wallis *H*_3_ = 8.361; 4 groups, *n* = 31, *p* = 0.0391). Dunn’s multiple comparisons test revealed a significant reduction in marble burying behavior in DSP4-treated rats compared to NaCl controls (*p* = 0.0347). All other comparisons were not statistically significant (*p* > 0.05). At 6 months, a two-way ANOVA was used to assess the effects of LPS, DSP4, and their interaction. A significant interaction effect was found (*F*_1,27_ = 6.785, *p* = 0.0148), while neither the main effect of DSP4 (*F*_1,27_ = 3.335, *p* = 0.0789) nor LPS (*F*_1,27_ = 8.59, *p* = 0.9927) reached significance. Tukey’s post hoc analysis revealed a significant reduction in marble burying in the DSP4 group without LPS compared to NaCl controls (*p* = 0.0230). No other group differences were significant (*p* > 0.05) (Marble hiding data visualized in Supplementary Fig. 1).

### Persistent motor deficits induced by loss of noradrenergic input

Motor performance was evaluated using the rotarod test. At 3 months the Kruskal–Wallis test indicated a highly significant difference among treatment groups (Kruskal–Wallis *H*_3_ = 23.42; 4 groups, *n* = 32, *p* < 0.0001). Dunn’s multiple comparisons test showed that both the DSP4 and DSP4-LPS groups had significantly reduced latency to fall compared to NaCl controls (*p* = 0.0020 and *p* = 0.0072, respectively). DSP4-treated rats also performed significantly worse than LPS-treated rats (*p* = 0.0020), and LPS-treated animals outperformed the DSP4-LPS group (*p* = 0.0072). Other comparisons did not reach statistical significance (*p* > 0.05) (Fig. 4c).

At 6-month point, two-way ANOVA showed no significant DSP4 × LPS interaction (*F*₁,₂₆ = 1.816, *p* = 0.1894) and no main effect of LPS (*F*₁,₂₆ = 1.648, *p* = 0.2106), but a strong main effect of DSP4 (*F*₁,₂₆ = 697.8, *p* < 0.0001). Tukey’s post hoc test revealed that DSP4-treated mice showed significantly reduced rotarod performance compared to NaCl controls (*p* < 0.0001). No significant difference was observed between LPS and NaCl (*p* = 0.2693), nor between DSP4 and DSP4-LPS (*p* > 0.9999). Both DSP4 and DSP4-LPS groups performed markedly worse than LPS (*p* < 0.0001) and NaCl (*p* < 0.0001) (Fig. 4d).

### Neuroinflammation is regionally selective and amplified by noradrenergic loss

**Fig. 5:**
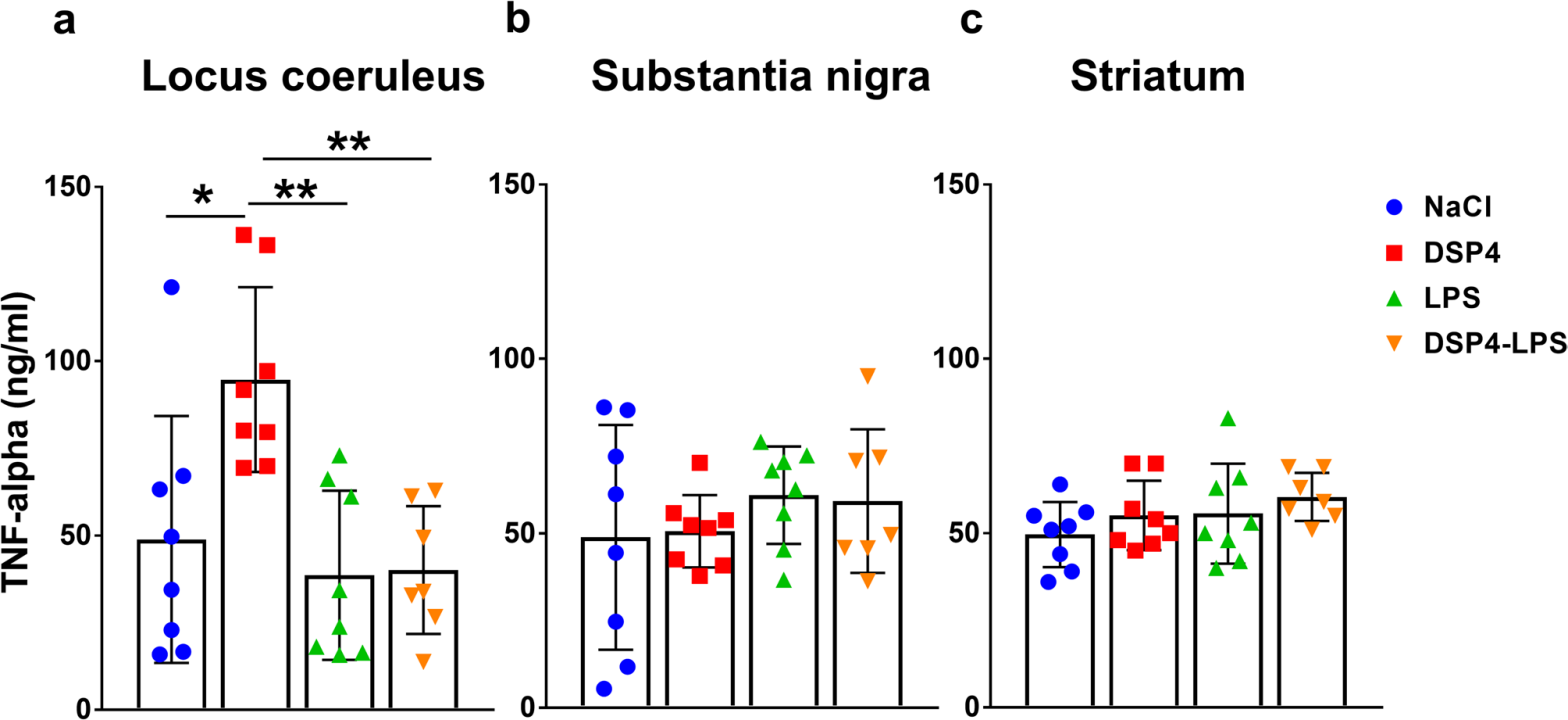
TNF-α expression. ELISA revealed increased TNF-α levels in the locus coeruleus of DSP4-treated animals at 3 months compared to NaCl, LPS, and DSP4-LPS, while no significant changes were detected in the striatum or substantia nigra. NaCl (*n* = 8), LPS (*n* = 8), DSP4 (*n* = 8), DSP4-LPS (*n* = 8). Bars represent mean ± SD. *p < 0.05, **p < 0.01.

Cytokine levels were assessed in the LC, SN and striatum to evaluate neuroinflammatory responses in these brain regions. At 3 months in the LC, two-way ANOVA revealed significant main effects for TNF-α for both DSP4-induced noradrenergic denervation (*F*₁,₂_7_ = 5.876, *p* = 0.0223) and LPS administration (*F*₁,₂_7_ = 11.07, *p* = 0.0025). Significant interaction between DSP4 and LPS was found (*F*₁,₂_7_ = 5.163, *p* = 0.0312), indicating synergistic effects. Post hoc Tukey´s test revealed that DSP4-treated animals had significantly elevated TNF-α levels compared to NaCl (*p* = 0.011), LPS (*p* = 0.002), and DSP4-LPS (*p* = 0.003) treatment groups (Fig. 5a). No significant changes in TNF-α were detected in the SN or striatum (Fig. 5b-c).

**Fig. 6:**
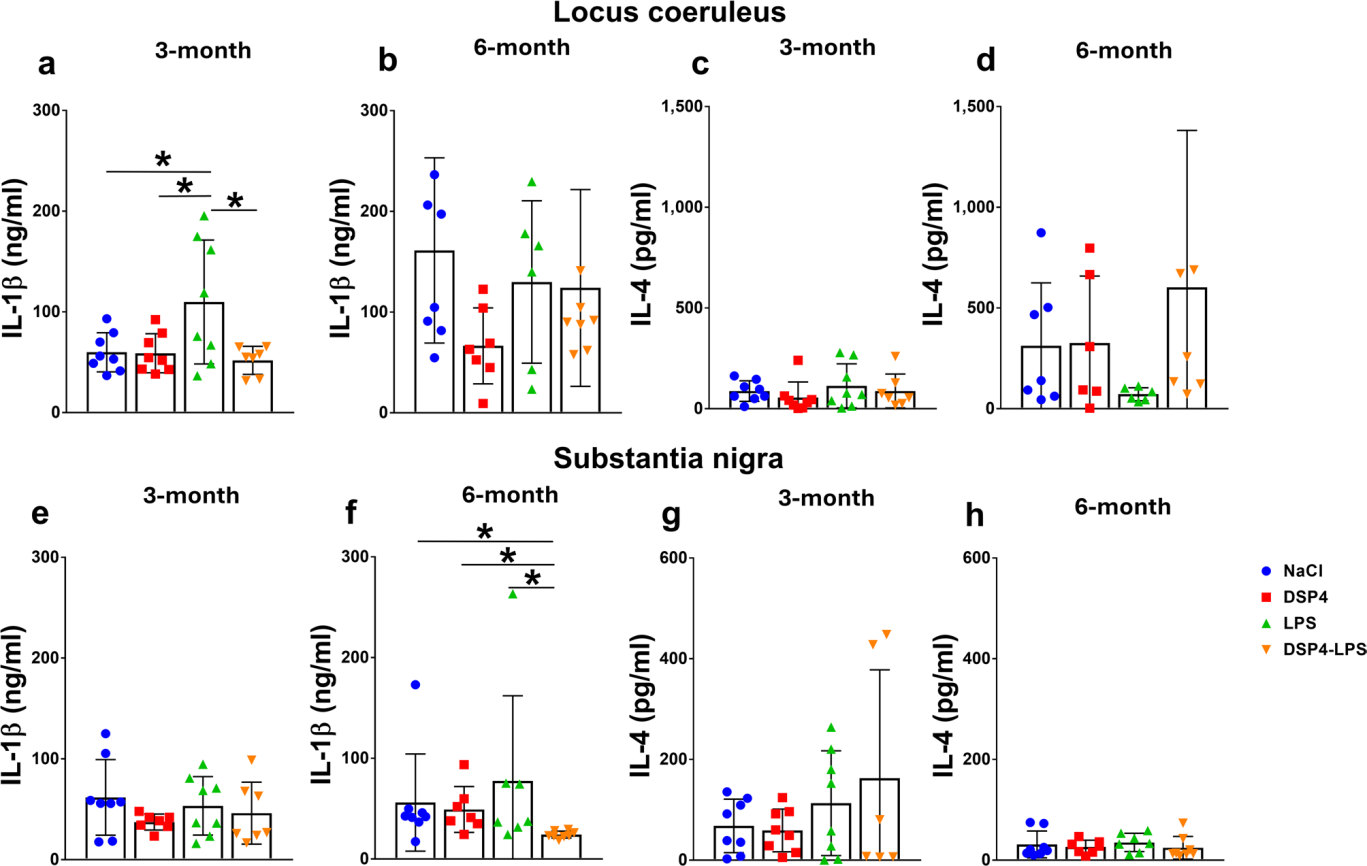
IL-1β and IL-4 expression. Locus coeruleus: At 3 months, IL-1β levels in the LC showed a significant interaction between LPS and DSP4 treatments. Post hoc comparisons revealed higher IL-1β expression in LPS-treated animals compared to NaCl, DSP4 and LPS-DSP4 (**a**). No significant differences in IL-1β levels were observed at 6 months (**b**). IL-4 levels in the LC were not significantly different at either 3 or 6 months, though the comparison between LPS and DSP4-LPS at 6 months trended toward significance (**c**-**d**). Substantia nigra: IL-1β levels at 3 months were not significantly affected by LPS or DSP4 treatments (**e**). At 6 months, IL-1β expression was significantly lower in the DSP4-LPS group compared to NaCl, DSP4, and LPS-treated groups (**f**). IL-4 levels in the SN showed no significant differences among treatment groups at either 3 or 6 months (**g**-**h**). NaCl (*n* = 8), LPS (*n* = 8), DSP4 (*n* = 8), DSP4-LPS (*n* = 8). Bars represent mean ± SD. *p < 0.05.

Additionally, IL-1β and IL-4 levels were measured in the LC and SN at 3 and 6 months to assess how immune responses in these regions are altered following an insult to the noradrenergic system. For the LC, IL-1β levels at 3 months were analyzed using two-way ANOVA. A significant main effect of LPS was observed (*F*_1,27_ = 5.514, *p* = 0.0264), while the main effect of DSP4 was not significant (*F*_1,27_ = 2.912, *p* = 0.0994). Additionally, there was a significant interaction between LPS and DSP4 (*F*_1,27_ = 5.157, *p* = 0.0313). Tukey’s post hoc analysis showed that LPS animals had significantly higher IL-1β expression than the other groups: NaCl (*p* = 0.0379), DSP4 (*p* = 0.0334), and DSP4-LPS (*p* = 0.0169) (Fig. 6a). At 6 months, no significant differences in LC IL-1β levels were detected (Kruskal–Wallis *H*_3_ = 5.28; *n* = 29, *p* = 0.1524), and post hoc tests showed no significant pairwise differences (*p* > 0.05) (Fig. 6b).

IL-4 expression in the LC did not differ among groups at 3 or 6 months (Kruskal–Wallis; 3 months: *H*_3_ = 3.261, *p* = 0.3532; 6 months: *D*_3_ = 6.339, *p* = 0.0962). Although the comparison between LPS and DSP4-LPS at 6 months approached significance (*p* = 0.0791), no pairwise comparisons were significant (*p* > 0.05) (Fig. 6c, d). In the SN, IL-1β levels at 3 months were analyzed using two-way ANOVA, which revealed no significant main effects of DSP4 (*F*_1,26_ = 0.0005, *p* = 0.9818) or LPS (*F*_1,26_ = 2.247, *p* = 0.1459), and no significant interaction between treatments (*F*_1,26_ = 0.661, *p* = 0.4237) (Fig. 6e). At 6 months, IL-1β expression in the SN showed significant group differences (Kruskal–Wallis, *H*_3_ = 12.05; 4 groups, *n* = 30, *p* = 0.0072). Post hoc Dunn’s tests indicated that the DSP4-LPS group had significantly lower IL-1β levels compared to NaCl (*p* = 0.0271), DSP4 (*p* = 0.0308), and LPS alone (*p* = 0.0339), while no differences were observed among the latter three groups (*p* > 0.9999) (Fig. 6f).

IL-4 levels in the SN were unchanged at both 3 and 6 months (Kruskal–Wallis, 4 groups, *n* = 30; 3 months: *H*_3_ = 0.2812, *p* = 0.9635; 6 months: *D*_3_ = 2.382, *p* = 0.4971). Post hoc tests confirmed no significant pairwise differences (*p* > 0.05) (Fig. 6g-h).

## Discussion

This study investigated the long-term effects of noradrenergic depletion on dopaminergic neuron survival in the SN under LPS-induced inflammatory conditions, alongside associated behavioral and cytokine alterations. Using a DSP4-induced lesion model of LC degeneration together with a low-dose LPS exposure, we demonstrate that a dual-hit consisting of induced chronic noradrenergic deficiency followed by an inflammatory insult exacerbates dopaminergic vulnerability, triggers behavioral deficits, and alters neuroinflammatory responses in a region-specific manner.

Consistent with previous findings^2^, our data show that DSP4 administration reduced TH-ir neurons in the LC and, over time, indirectly affected dopaminergic neurons in the SN, with effects nearing statistical significance. This contrasts with earlier studies^31–33^ that reported minimal effects on SN neurons, likely due to their focus on short-term noradrenergic denervation. In contrast, our findings suggest that chronic noradrenergic depletion contributes to progressive dopaminergic degeneration in the SN, underscoring the importance of chronic models when investigating Parkinson’s disease pathology as well as support the Braak hypothesis, which posits that early lesions in vulnerable brainstem regions such as the LC may predispose interconnected regions like the SN to subsequent degeneration^1^.

Regarding DSP4 specificity, prior work confirms that this neurotoxin primarily targets noradrenergic neurons with neglectable direct impact on dopaminergic systems^16,34,35^. Our previous findings align with these observations, suggesting that the dopaminergic degeneration arises not from direct DSP4 toxicity, but from the chronic loss of noradrenergic modulation and associated microglial activation^2^.

A direct inflammatory challenge with LPS further reduced TH-ir neurons in both the LC and SN, supporting previous reports that systemic inflammation promotes nigral vulnerability^36^. Importantly, the use of a low LPS dose allows us to reveal additive vulnerabilities without overwhelming the system, a key consideration for modeling subtle inflammatory contributions to neurodegeneration. The findings of this study provide evidence in support of the dual-hit hypothesis. Specifically, when the SN dopaminergic system is already compromised, as herein through the loss of noradrenergic input, it exhibits increased susceptibility to secondary challenges such as inflammatory insults. Accordingly, animals treated with both DSP4 and LPS exhibited significantly greater SN degeneration than those receiving DSP4 alone, consistent with findings from a previous study in mice^18^. The findings underscore the importance of noradrenergic integrity in maintaining brain resilience and health, and in protecting dopaminergic neurons against insults, consistent with emerging multi-hit models of Parkinson’s disease pathogenesis^37–39^. Although, it should be noted that LPS alone also significantly reduced SN dopaminergic cell survival, and that the additional degeneration observed in the DSP4–LPS group may reflect the cumulative impact of dual insults. This cumulative effect suggests a process that goes beyond the gradual neuronal loss of normal aging, representing instead a pathologically accelerated degeneration; in this vulnerable state, an additional challenge such as a CNS infection could drive the system past a critical threshold, precipitating the emergence of Parkinsonian symptoms.

As for cytokine alterations, profiling revealed notable region- and time-specific changes. TNF-α levels were significantly elevated in the LC at 3 months following DSP4 treatment, indicating localized neuroinflammatory activation. In contrast to findings from a mouse study^40^, TNF- α levels in the SN remained largely unchanged. Interestingly, IL-1β levels in the LC decreased after DSP4 treatment, a finding that contrasts with the traditional expectation of heightened pro-inflammatory signaling following neuronal injury. This reduction may reflect a compensatory, anti-inflammatory shift in microglial phenotype or altered β-adrenergic regulation of cytokine production, as suggested by previous work on noradrenergic control of microglial function^41–43^. The timing of cytokine measurement is critical, as IL-1β expression may initially rise and later decline as a feedback response^30,44^.

These findings imply that noradrenergic depletion disrupts normal cytokine response in the SN, potentially impairing appropriate immune surveillance and exacerbating neurodegenerative processes. The complex interplay between TNF-α elevation in the LC and IL-1β suppression in the SN suggests that LC- noradrenergic neurons play a nuanced role in regulating neuroinflammatory dynamics.

Behaviorally, DSP4 treatment induced significant anxiety-like behavior and motor dysfunction, consistent with the critical role of the LC in modulating emotional and motor circuits. Elevated anxiety in the zero-maze at 6 months highlights the non-motor symptom burden associated with early LC degeneration in Parkinson’s disease. While marble burying behavior was also increased, the specificity of this test for anxiety-like behavior is debated, as it may also reflect exploratory or compulsive behaviors^45,46^. Additionally, although some significant findings emerged, interpreting the data was challenging due to high variability and large standard deviations. Therefore, the model would benefit from further studies with a larger sample size. Nonetheless, the combined evidence of behavioral impairments and neuropathological changes enhances the translational relevance of our model.

Collectively, these results emphasize the importance of noradrenergic integrity in maintaining dopaminergic neuron resilience and regulating neuroinflammatory processes. Our findings align with growing evidence that early LC pathology contributes to Parkinson’s disease progression. Future studies are desired to explore the temporal dynamics of immune activation and examine whether restoring noradrenergic tone can mitigate neurodegeneration and behavioral decline.

Given the rising prevalence of Parkinson’s disease and the limited efficacy of current dopaminergic therapies in addressing non-motor symptoms, focusing on noradrenergic modulation offers a promising new direction. Investigating therapeutic agents that preserve LC function or mimic noradrenergic immunomodulatory effects could yield significant advances in delaying disease progression and improving quality of life for affected individuals.

## Conclusion

These findings support the dual-hit hypothesis, in which multiple converging insults drive neurodegeneration, with numerous factors being critical for maintaining neuronal resilience. The study highlights a plausible neurodegenerative process, emphasizing the essential neuroprotective role of the noradrenergic LC in preserving dopaminergic neuron integrity within the SN. Behavioral assessments revealed persistent motor deficits and increased anxiety-like behaviors in DSP4-treated animals, highlighting the broader impact of LC dysfunction on both motor and non-motor domains. Region-specific alterations in cytokine profiles, notably the upregulation of TNF-α within the LC and the modulation of IL-1β expression in the SN further emphasize the critical interplay between noradrenergic signaling and neuroimmune regulation.

Overall, these results advance our understanding of the early pathological mechanisms contributing to Parkinson’s disease and reinforce the potential of targeting the noradrenergic system as a therapeutic strategy to delay disease progression and mitigate both motor and non-motor symptoms.

## Supplementary Information

Below is the link to the electronic supplementary material.

Supplementary Material 1

Supplementary Material 2

## Data Availability

Upon reasonable request, the data supporting this study can be provided by the corresponding author.

## Abbreviations

LC: Locus coeruleus
SN: Substantia nigra
NA: Noradrenaline
DSP4: N-(2-chloroethyl)-N-ethyl-2-bromobenzylamine hydrochloride
LPS: Lipopolysaccharide
TH: Tyrosine hydroxylase
TH-ir: Tyrosine hydroxylase immunoreactive
ELISA: Enzyme-linked immunosorbent assay
IL-4: Interleukin-4
IL-1β: Interleukin-1 beta
TNF -α: Tumor necrosis factor alpha
NaCl: Sodium chloride
