# Supplementary figures and images for: Synergetic effect of a DSP4-induced locus coeruleus lesion and systemic LPS exacerbates substantia nigra dopaminergic neuron loss
Source: Sci Rep. 2025 Dec 24;15:44638. doi: 10.1038/s41598-025-33147-8 (PMC12749801; doi:10.1038/s41598-025-33147-8)

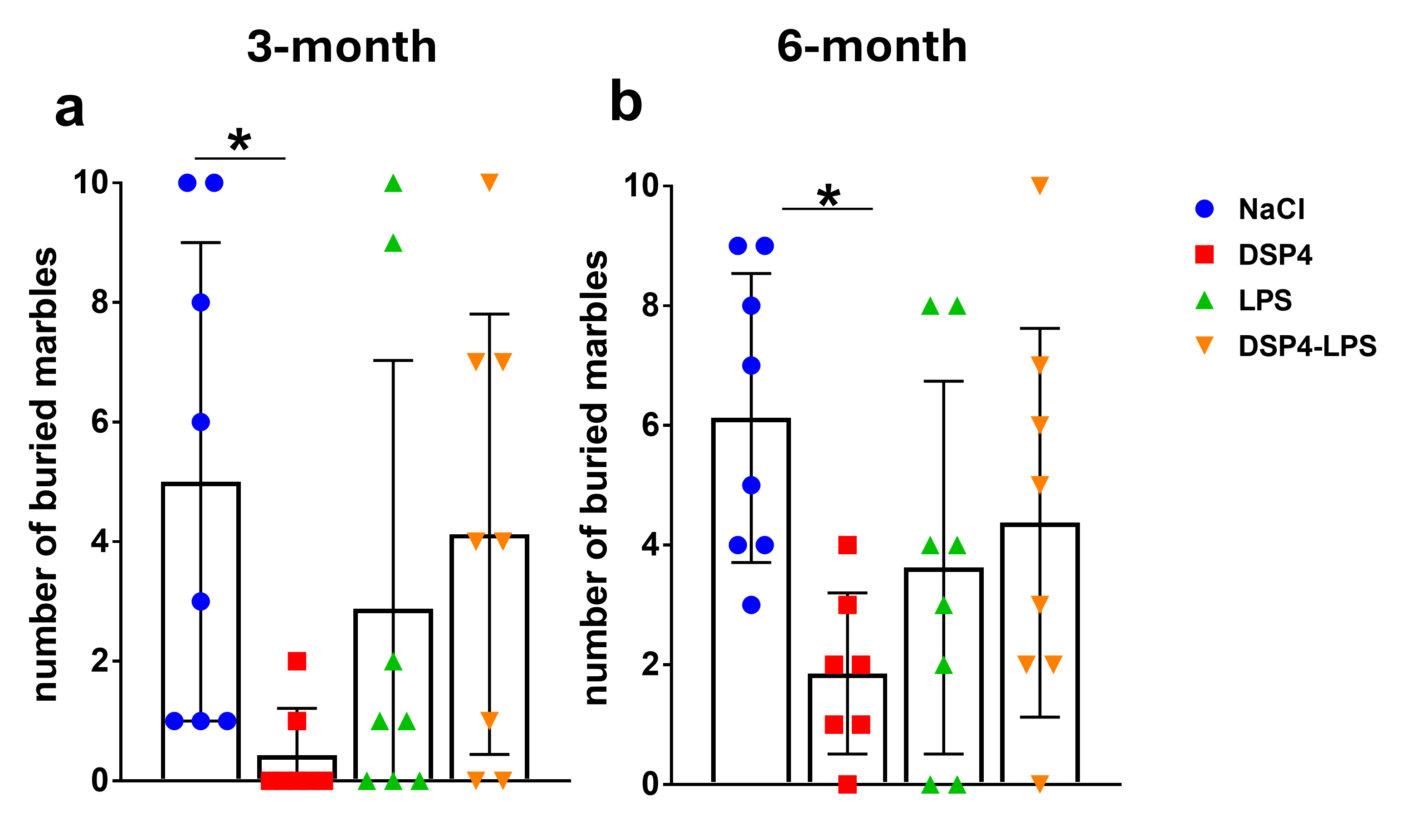

Supplement: Supplementary file 1 — Supplementary Material 1 [file 41598_2025_33147_MOESM1_ESM.tif]
