# Supplementary material for: Synergetic effect of a DSP4-induced locus coeruleus lesion and systemic LPS exacerbates substantia nigra dopaminergic neuron loss
Source: Sci Rep. 2025 Dec 24;15:44638. doi: 10.1038/s41598-025-33147-8 (PMC12749801; doi:10.1038/s41598-025-33147-8)
Supplement: Supplementary file 2 — Supplementary Material 2 [file 41598_2025_33147_MOESM2_ESM.docx]

**Supplementary Figure 1**. Marble-burying behavior. DSP4-treated animals without LPS exhibited significantly increased marble-burying activity compared to controls at 6 months (a, b). However, the results showed a wide variation among individuals and are hard to interpret.

NaCl (n = 8), LPS (n = 8), DSP4 (n = 8), DSP4-LPS (n = 8). Bars represent mean ± SD. **p* <0.05

**Article title:** Synergetic effect of a DSP4-induced Locus Coeruleus Lesion and Systemic LPS exacerbates Substantia Nigra Dopaminergic Neuron Loss

**Journal name:** Scientific Reports

**Author names:** Feryal Şimşek, Diana Papová, Emilia Henriksson, Sonia Olmedo-Díaz, Marcus Blyberg, Roine El-Habta, Ana Virel, Sara af Bjerkén

**Corresponding Author:** Sara af Bjerkén, email: [sara.af.bjerken@umu.se](mailto:sara.af.bjerken@umu.se)

Department of Medical and Translational Biology, Umeå University, Umeå, Sweden, Department of Clinical Sciences, Neurosciences, Umeå University, Umeå, Sweden
